# 引言

**DOI:** 10.3724/SP.J.1123.2021.06004

**Published:** 2021-08-08

**Authors:** Lingxin CHEN, Jinhua LI

**Affiliations:** 中国科学院烟台海岸带研究所

近年来,环境污染问题不断加重,对生态平衡和人类健康造成了巨大威胁,引起了全球各国的广泛关注。我国对环境安全和污染问题高度重视,连续出台系列举措,“生态文明建设”和“绿水青山就是金山银山”等绿色环保理念深入人心。虽然我国的环境污染问题初步得到有效遏制,但陆海环境污染物种类繁多、环境基质复杂和污染隐蔽性等问题仍然突出,特别是在“陆海统筹,保护优先”的新格局下,陆源生产造成的各类污染及海洋环境污染等状况更加呼唤环境污染物快速、灵敏的分离分析方法与技术。

创新分离分析方法与技术,对含量低、危害大的环境污染物,尤其是微纳塑料、抗生素等各类新型污染物进行精准测定,是评估环境质量、监控和修复陆海环境、确保环境安全的重要基础。通常,污染物在环境介质中处于痕量、超痕量水平,存在种类繁多、成分和基质复杂、理化性质各异、危害大等特点,这就要求分析方法具有灵敏、准确、简便、快速,甚至连续自动等优势。色谱及其相关分离分析方法与技术具有优异的分离性能,在分离复杂样品多组分目标物方面潜力巨大。化学测量学、环境科学、材料科学等学科的深度交叉融合,及仪器技术、材料技术等的迅猛发展、交叉应用,进一步提升了色谱分离分析的性能, 推动了色谱及相关技术在环境分析领域中越来越广泛的应用,为污染控制、溯源和风险评价等提供了重要的理论和技术支撑。

推出本专刊,旨在介绍我国在色谱分离分析污染物的方法与技术领域的一些研究前沿及进展,帮助《色谱》读者快速了解和把握我国乃至全球此领域研究的概况。同时,希望本专刊文章能对读者有所帮助或启发,提高我国在本领域的研究水平。经过专家严格评审,共刊出7篇研究报告和8篇综述。在此,感谢各位作者踊跃投稿,也敬请各位读者批评指正。

中国科学院烟台海岸带研究所

本专刊客座主编

陈令新 研究员 李金花 副研究员


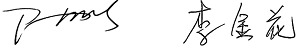


2021年6月2日

